# Reduction of adult employees’ anxiety disorder in the work environment: Implications for anthropological philosophers and a case of multivariate analysis

**DOI:** 10.1097/MD.0000000000045834

**Published:** 2026-01-16

**Authors:** Sebastian Okechukwu Onah, Queen E. Igabari, Uchechukwu Hope Ekwueme, Kelechi Ruth Ede, Fedrick C. Onah, Moses Onyemaechi Ede, Ifeyinwa Manafa, Ogochukwu Vivian Nwabuani, Obiageli Loretta Aniaku, Michael Chugozie Anyaeihe, George Ohabuenyi Abah, Innocent I. Enweh, Monday Ume Nwodo, Ndubuisi Collins Enyi

**Affiliations:** a Department of Sociology and Anthropology, University of Nigeria, Nsukka, Nigeria; b Department of Mathematics, Faculty of Science, Delta State University, Nigeria; c Department of Educational Foundations, Faculty of Educations, University of Nigeria, Nsukka, Nigeria; d Department of Agricultural Education, Faculty of Vocational and Technical Education, University of Nigeria, Nsukka, Nigeria; e Department of Educational Psychology, Faculty of Education, University of Johannesburg, Johannesburg, South Africa; f Department of Educational Foundations, Faculty of Educations, Chukwuemeka Odumegwu Ojukwu University, Anambra State, Nigeria; g Department of Adult Education, Faculty of Educations, University of Nigeria, Nsukka, Nigeria; h Department of Science Education, Faculty of Educations, University of Nigeria, Nsukka, Nigeria; i Department of Philosophy, University of Nigeria, Nsukka, Nigeria; j Alex Ekwueme Federal University Teaching Hospital, Abakaliki, Nigeria; k St. Augustine Catholic Church, Manso Amenfi, Ghana.

**Keywords:** anxiety disorder, CBT, cognitive behavioral therapy, employees

## Abstract

**Background:**

According to the literature, people who suffer from financial anxiety exhibit denial, skewed financial actions, and cognitive disturbances related to debt. As a result, the purpose of this study was to determine the effect of cognitive behavioral financial treatment on loan anxiety among employees.

**Methods:**

A group-randomized trial design was adopted, with 106 public servants randomly allocated to the intervention group and a waitlist control group as participants. Two versions of the Financial Anxiety Scale (FAS-A and FAS-P) created by 2 authors were utilized to collect data. Intervention consisted of a 3-month cognitive behavioral financial treatment program presented in 5 sessions, with the goal of assisting participants in identifying and changing their dysfunctional beliefs regarding the loan and other money-related concerns in order to bring about adaptive emotional and behavioral change.

**Results:**

A 2-way analysis of covariance demonstrated a consistent decrease in the mean financial anxiety scores of employees on loan in the treatment group over time, as measured by FAS-A and FAS-P.

**Conclusion:**

Based on the findings, we recommend that an intervention program be developed to identify and diagnose distorted or maladaptive financial attitudes and behavior in people suffering from various debt-related mental health disorders.

## 1. Introduction

Anxiety disorder is characterized by feelings of stress, concerned thoughts, and physical changes such as elevated blood pressure.^[1]^ Anxiety is defined as a person’s apparent incapacity to predict, manage, or achieve the desired outcome when confronted with a threat,^[2]^ as well as sensations of tension and worrisome thoughts. Anxiety can also cause muscle tension, trembling, sweating, shaking, muscle discomfort, and neurological system difficulties.^[3–5]^ However, if it becomes severe or begins to interfere with one’s daily functioning, it might be classified as a condition or illness.^[6]^

Anxiety is one of the most prevalent public mental health problems,^[7,8]^ causing considerable negative impacts on the quality of life^[9,10]^ that cut across ages.^[11]^ Globally, it was estimated that about 272.2 million people had an anxiety disorder^[12]^ among which adults aged 20 to 64 years were shown to have the highest prevalence.^[13]^ However, reports from countries on the occurrence of adult anxiety vary. Reports show that about 1.7% of adults suffer from an anxiety disorder in Malaysia.^[14]^ In Europe, evidence showed that the most vulnerable population is the adult population.^[15]^ In fact, about 70.1 million of Europe’s population is grossly affected by an anxiety disorder.^[15]^

Like other countries, Nigerian studies also documented a fluctuating prevalence of anxiety disorder following research evidence. For instance, 20.9% prevalence of anxiety among adults was reported in 2018 using Lagos State Mental Health Survey.^[16]^ In 2017, the prevalence of anxiety disorder among 425 adult patients in Nigeria was found to be high.^[17–20]^ By this statistical evidence, we argued that anxiety disorder occurs in adults compared to other populations.

Adults with anxiety disorder tend to be vulnerable to psychological factors that are associated with increased risks of trauma and financial incompetence. Among the factors are substance use and exposure to violence and traumatic events.^[21]^ Evidence from sub-Saharan African countries shows that poverty-related factors include lower socioeconomic status,^[22]^ lack of social capital support, overdependence,^[23,24]^ and financial incompetence as a source of anxiety.^[25]^ However, the cognitive ability of individuals with financial anxiety to cope with the current financial situation in Nigeria is essential. Instead, individuals with financial anxiety display poor financial competence widespread disbelief, and cognitive disturbance about fundamental economic concepts^[26,27]^ and distorted financial behaviors.^[28,29]^

Cognitive disturbance and distorted financial behaviors tend to be more prevalent among employees, particularly in the Nigerian context. They seem to encounter a series of complex financial incompetencies in their decisions as they determine how to fund their bills such as utilities, school fees, family upkeep, health challenges, commitments, and compulsive shopping, among others. Hence, they tend to have more pressure, more burnout, and a greater prevalence of anxiety than the general population who in one way or another are dependent on them.^[30–32]^ The combined result is that employees involved find themselves in substantial debt that can leave them seriously indebted for years^[33,34]^ and tend to expose them to obtaining loans from micro-finance or commercial banks.

To adult employees, loan indebtedness is associated with negative psychological repercussions including a decreased sense of ability to manage one’s money, lower self-esteem, decreased sense of financial wellbeing, lower productivity, and higher levels of overall stress, and anxiety.^[35,36]^ In organizational setting, indebtedness has been known to impair job performance among employees.^[37,38]^ It is found to be responsible for high rates of sick leave, absenteeism, and low staff turnover.^[39]^ Consequently, we claim that indebted adult employees tend to have unrealistic and dysfunctional cognitive and behavioral responses in the workplace.

Empirical studies found that personal debt is a strong predictor of anxiety,^[40]^ depression, and general psychological distress.^[41]^ These findings highlight the psychological potency of being indebted and, as others have noted, have implications for psychological and health consequences on the debtors.^[42–44]^ Hence, the need for a cognitive-behavioral-oriented approach that will help reduce the financial anxiety among adult employees on loans. However, to date, there is a paucity of empirical-based interventions addressing anxiety disorders, particularly among adult employees on loan in developing countries like Nigeria. In view of that, studies have suggested and recommended cognitive-behavioral approaches for addressing clinical (e.g., depression) and nonclinical (e.g., anxiety and stress) problems^[45–47]^ which involves the exploration of thinking, feeling, and behaving, with cognitions and behaviors being the main focus^[48]^ for reduction of anxiety across populations.

Cognitive behavioral therapy (CBT)^[48]^ proposed the basic premise that mental disorders and psychological distress are maintained by cognitive factors. The CBT approach, as pioneered by Beck^[49]^ holds that maladaptive cognitions contribute to the maintenance of emotional disturbance and behavioral problems. Hence, CBT focuses on challenging and changing unhelpful cognitive distortions (e.g., negative views about self, future, and the world) and dysfunctional behaviors by improving emotional regulation.^[50,51]^ This is because CBT is based on the belief that maladaptive behaviors are the product of thought distortions.^[52]^ According to Beck model, these maladaptive cognitions include general beliefs, or schemas, about the world, the self, and the future, giving rise to specific and automatic thoughts in particular situations.

The overall goal of treatment is symptom reduction, improvement in functioning, and remission of the disorder. In order to achieve this goal, the participant becomes active in a collaborative problem-solving process to test and challenge the validity of maladaptive cognitions and to modify maladaptive behavioral patterns. CBT is a “problem-focused” and “action-oriented” form of therapy, meaning it is used to treat specific problems related to a diagnosed mental disorder. The therapist’s role is to assist the client in finding and practicing effective strategies to address the identified goals and decrease symptoms of the disorder.^[53]^

CBT includes a number of cognitive or behavior psychotherapies that treat defined psychopathologies using evidence-based techniques and strategies.^[54]^ The strategy integrates elements of various therapeutic approaches that are regularly used by mental health professionals. One of them is cognitive behavioral financial therapy (CBFT).^[55]^ All of these therapies are blendings of cognitive and behavior-based elements that concentrate on changing unhelpful habitual responses and reinforcing positive behaviors. However, the present study focused on CBFT. This is geared towards the notion that the observed anxiety disorder and other mental health problems experienced by employees on loan and their financial attitudes and behaviors are functions of more than just a lack of financial acuity. Hence, they have been at a loss as to how to provide meaningful guidance to them.

CBFT is an extension of CBT developed by Nabeshima and Klontz.^[55]^ This technique is used by advisors and financial therapists to help people reassess self-defeating beliefs that hinder or impede positive financial behavior. The goal of the technique is to help people experiencing adverse financial conditions, create a mental mindset to cope with their challenging situations and develop proactive strategies and behaviors to address, resolve, and improve their circumstances.^[56]^

The techniques used in CBFT are restructuring cognitive beliefs and coping strategies. In restructuring cognitive beliefs, clients with dysfunctional cognitive distortions who represent thinking that is generally not true to reality and that which adversely encourages behavior that prevents people from succeeding financially are restructured by financial therapists. The process of restructuring dysfunctional financial beliefs is generally completed in a series of steps which include identifying irrational beliefs, challenging irrational beliefs, testing the validity of irrational beliefs, creating replacement beliefs, and modifying behavior.^[56,57]^ The overall process is problem-focused and generally deals with the present rather than the past. In the second technique, no attempt is made to change negative cognitive beliefs, but rather these beliefs are acknowledged as real, and coping strategies are used to deal with them.^[58,59]^

Given the high prevalence rate of anxiety disorder attributable to adult employees with loans and possible intervention recommended by previous studies, the present study investigated the impacts of CBFT on anxiety among employees on loans. Therefore, we hypothesized that cognitive-behavioral financial therapy would be significantly impactful in reducing anxiety disorder among employees on loan who were exposed to CBFT program compared to the comparison group. We also hypnotized that there will be a sustained significant impact of CBFT on anxiety among employees on loan at follow-up stage.

## 2. Methods

### 2.1. Ethical consideration and compliance

The researchers sought for ethical approval from the Research Ethics Committee of the Faculty of Education, University of Nigeria, Nsukka, and the approval was granted for the study. Official permission to conduct the study was granted by the microfinance banks. Finally, a letter of informed consent was obtained from the employees on loan who showed willingness to participate in the study after a brief explanation of the objectives of the study. This research was conducted in accordance with the research principles of the American Psychological Association and registered after completion on July 19, 2020 with Clinical trial registration number UMIN000041131 at the UMIN Clinical Trials Registry.

### 2.2. Participants

A total of 106 civil servants were used as participants. The power of the sample was ascertained using G-Power statistical software. The participants’ demographic characteristics are given in Table 1.

**Table 1:** Demographic characteristics of the participants.

Characteristics	CBFT group,n (%)	Waitlist control group, n (%)
Gender		
Male	30 (56.6)	29 (54.7)
Female	23 (43.4)	24 (45.3)
Age (yr)		
30–40	20 (37.7)	25 (47.2)
41–50	21 (39.6)	19 (35.8)
51 and above	12 (22.7)	9 (17.0)
Relationship status		
Single	16 (30.3)	12 (22.6)
Married	28 (52.8)	37 (69.8)
Separated/divorced	4 (7.5)	3 (5.7)
Widow	5 (9.4)	1 (1.9)
Length of loan (LOL) (yr)		
1–2	18 (34.0)	26 (49.1)
3–4	22 (41.5)	16 (30.2)
5 and above	13 (24.5)	11 (20.7)
Education		
NCE	3 (5.7)	6 (11.3)
BD	20 (37.7)	14 (26.4)
MD	17 (32.1)	17 (32.1)
PhD	13 (24.5)	16 (30.2)
Civil service		
Police officers	11 (20.8)	10 (18.9)
Lecturers	13 (24.5)	17 (22.1)
Sec. sch. teachers	11 (20.8)	9 (17.0)
LG employees	9 (17.0)	7 (13.2)
Nurses	9 (17.0)	10 (18.9)
Length of employment (yr)		
1–10	25 (47.2)	21 (39.6)
11–12	16 (30.2)	16 (30.2)
21 and above	12 (22.6)	16 (30.2)
Gross monthly income/salary		
≤$150	14 (26.5)	12 (22.6)
$151–200	19 (35.8)	20 (37.8)
≥$201 and above	20 (37.7)	21 (39.6)
Addiction profile		
Drug dependence	17 (32.1)	15 (28.3)
Alcohol dependence	14 (26.4)	17 (32.0)
Gambling	10 (18.9)	11 (20.8)
Compulsive buying	12 (22.6)	10 (18.9)

CBFT = cognitive behavioral financial therapy, n = number of participants.

### 2.3. Design

The design adopted for this study is a group-randomized trial design. Group-randomized trial design according to Murray^[60]^ helps experimenters to assign participants into groups (treatment and comparison groups). Group-randomized trial design encourages randomization; hence it ensures a greater possibility of equivalence.^[61]^ This is because the design will enable the researchers to randomly assign participants to experimental and control groups.

### 2.4. Measures

A number of measures were used in this study including Financial Anxiety Scale (FAS) and Money Attitude Scale. These are described below.

#### 2.4.1. Financial Anxiety Scale

FAS-A (a 7-item scale developed by Archuleta et al)^[62]^ was used to assess one’s current self-reported level of financial anxiety. Participants indicated the extent to which they agree or disagree with each statement on a 7-point scale of Never = 7, Rarely = 6, Occasionally = 5, Sometimes = 4, Frequently = 3, Usually = 2 to Always = 1. Once items were summed, scores on the FAS-A can range from 7 to 49. Some of the items on the scale include: “*I feel anxious about my financial situation*,” “*I have difficulty sleeping because of my financial situation*,” and “*I am irritable because of my financial situation*.” The items on this measure were averaged to get a mean score. In the study, participants’ scores ranged from 1 to 7; higher total scores indicated higher levels of financial anxiety, whereas lower scores indicated lower levels of financial anxiety. The Archuleta et al^[62]^ version of FAS-A has been validated and shown to be reliable (α = 0.94) in a sample of college students at a Midwestern university’s peer financial counseling center. Faison^[63]^ found the reliability indices of FAS-A using Cronbach alpha to be 0.946. In another study, Archuleta et al^[62]^ found the internal reliability of FAS-A to be high (α = 0.94). Faison^[63]^ found the Cronbach alpha for the FAS of young adults to be α = 0.946. Grable et al^[64]^ found the Cronbach alpha of FAS-A to be 0.95. The Cronbach alpha for the FAS in the current study of employees on loan is α = 0.86.

A second version of the Financial Anxiety Scale (FAS-P) is a 10-item scale developed by Prawitz et al^[65]^ to measure participants’ anxious disposition toward cognitive engagement with one’s personal finances on a 4-point Likert-type scale. The scale ranged from very true = 1, somewhat true = 2, somewhat untrue = 3 to completely untrue = 4 where lower numbers indicate a higher level of financial anxiety. Some of the items on the scale include: I find monitoring my bank or credit accounts very boring, thinking about my personal finances can make me feel guilty, I do not make a big enough effort to understand my finances, I find looking at my bank statements unpleasant, Thinking about my personal finances can make me feel anxious, I get myself into situations where I do not know where I’m going to get the money to “bail” myself out, etc. The reliability indices α = 0.809 were established using Cronbach alpha.^[65]^ The present study found the reliability indices of α = 0.855. In this study, we used FAS-A to represent the FAS developed by Archuleta et al^[62]^ and FAS-P for the FAS developed by Prawitz et al.^[65]^

### 2.5. Procedure

**Figure 1.**
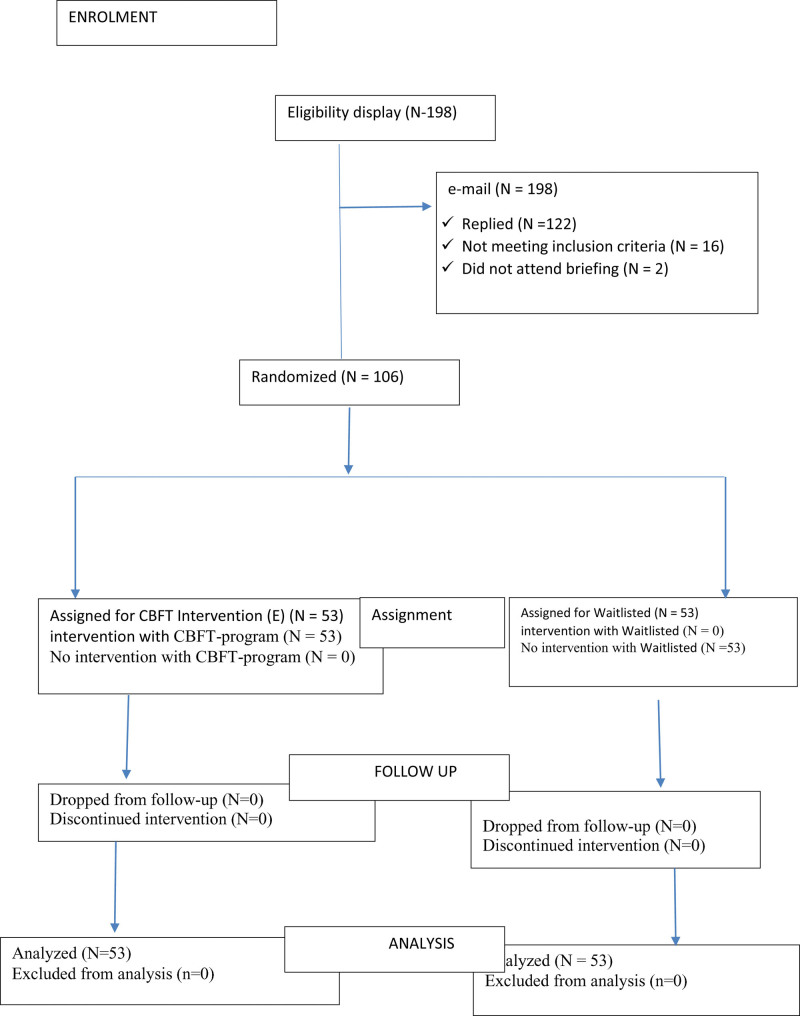
Participants’ eligibility criteria using CONSORT flow diagram. CBFT = cognitive behavioral financial treatment, CONSORT = Consolidated Standards of Reporting Trials.

The researchers sought and got the e-mail addresses and phone numbers of 198 employees on loan from microfinance banks in March to May 2019, and a consent letter was sent to each of them through SMS and mail. A total of 122 indebted employees replied to the sent SMS and mail and were followed up by the researchers to determine the inclusion criteria. The inclusion criteria are: must have been indebted for 12 months and beyond, the loan must be domiciled in a microfinance bank, must be a civil servant, appointment must be confirmed by the employer, must be an attempted or married employee, and must show wiliness to participate till the end of the program. The participants who did not meet with inclusion criteria were excluded based on the following exclusion criteria: any indebted employee receiving treatment from helping professionals, any indebted employee who does not complete and return the written informed consent, an indebted employee whose loan is domiciled in commercial banks, and indebted employees whose loan duration is below 1 year. Out of 122 indebted employees, 108 employees who met the study inclusion criteria were invited for briefing and randomly assigned into groups at Enugu State. Of the 108 participants invited, 106 attended the meeting and were later assigned into 2 groups. Thus, 53 participants were assigned to the experimental group and 53 participants were assigned to the waitlist control group using a simple random allocation sequence with the help of random allocation software (see Fig. 1). This was achieved by inscribing “E” and “W” (depicting experimental and waitlisted control groups, respectively) in a paper in duplicated forms of 53 each. The papers were thereafter folded and placed inside a container and the participants were allowed to pick. Groups were assigned to them based on their pickings. That is, 53 participants were assigned to the CBFT group while 53 participants were also assigned to a waitlist control group (see Saghaei)^[66]^ (see Fig. 1). Participants in the CBFT group were later divided into 2 subgroups (first group = 27 participants, second group = 26 participants). Participants in the CBFT group and waitlisted comparable groups were measured using instruments to ascertain the baseline of their financial anxiety.

The participants in the CBFT group were given CBFT program by cognitive behavioral financial therapists and counseling psychologists whose focuses were on helping the participants identify and change their dysfunctional beliefs about loans and other money-related issues to bring about adaptive emotional and behavioral changes. The intervention was implemented using the English language. The therapists have basic training in cognitive-behavioral approaches and counseling psychology practiced for more than 10 years. Approximately, CBFT program lasted for 3 months (July–September 2019). Each therapy session lasted for 90 minutes per 2 weeks. The participants in the waitlisted control group did not receive the CBFT program until after Time 1, Time 2, and Time 3.

#### 2.5.1. Session 1: Establishment of therapeutic alliance and identifying irrational beliefs

The therapists started by establishing a strong therapeutic alliance with the participants in the CBFT group wherein they demonstrated a sense of competence and care, showed warmth and positive regard, and empathized with the participants on the economic situation of the nation. Thereafter, the therapists shared the agenda of the session. The participants were asked to state situations that have triggered their maladaptive feelings and thought patterns about being indebted. Among the errors as identified by the participants include: since I got involved in a loan, I can never achieve what my colleagues have achieved, I feel ashamed if my friends discover that I owe the bank, ever since I got indebted, I have lagged behind in meeting my obligations as the head of my household; my indebtedness makes me look inferior among my equals; I find monitoring my bank or credit accounts very boring knowing full well that it will not surmount my financial obligation, I have difficulty paying bills because of not enough income among others. The therapists focused on helping the participants identify their errors in thinking in response to the identified money script preponderance.

#### 2.5.2. Section 2: Challenging irrational beliefs and collaboration between therapist and participants

Here the therapists focused on challenging the identified irrational beliefs, helping participants explore the evidence confirming or refuting the beliefs to help them deescalate the intense effects of such beliefs on them. Some of the errors as identified by the participants and their matching challenge from the therapists include: *error*: “since I got involved in loan, I can never achieve what my colleagues have achieved,” *challenge*: “but mind you that those of your colleagues whom you look up to did not achieve what you used the loan to achieve, or have you forgotten?”; *error*: “I feel ashamed if my friends discover that I am owing bank, ever since I got indebted,” *challenge*: “why living your life for others? ‘Be mindful of things that are of more important to your welfare than paying attention to what others think about you’, after all you have your live to lead”; *error*: “I have lagged behind in meeting my obligations as the head of my household,” *challenge*: “you can make your household see reasons with you, since the essence is for the progress of the entire household”; *error*: “my indebtedness makes me look inferior among my equals,” *challenge*: “why the broadcast about your indebtedness? You can keep it to yourself and politely cut down on frivolities”; *error*: “I feel worried seeing my salary alert knowing full well that it will not surmount my financial obligation,” *challenge*: “then remove alert notification.”

The therapists also gave out varieties of cognitive and behavioral coping strategies that could help the participants challenge the irrational thoughts as they creep up. Among the strategies are thought-stopping prompts, signs, cues, and imageries (e.g., interrupting negative self-talk by visualizing a STOP sign and “yelling” internally, “STOP”), using a cue card to help them replace irrational beliefs with more accurate scripts (e.g., several helpful statements written on a card to be kept in wallets or bags for reference, such write-ups as: “downfall of a man is not the end of his life,” “there is no harm in trying,” “it will end in praise,” “when there is life, there is hope,” keep the candle burning at all cost, etc), and deep diaphragmatic breathing to decrease anxiety.

#### 2.5.3. Section 3: Testing the validity of beliefs and a focus on the present

Having identified and challenged the automatic thoughts, the therapists introduced a number of strategies to test and identify which ones are incorrect, unhelpful, or self-defeating. Firstly, the therapists gave the definition of belief terms (e.g., anxiety, indebtedness, financial success) to the participants so they could sufficiently understand it for proper evaluation. In collaboration with the participants, the therapists examined the evidence supporting and contradicting the identified beliefs about being indebted. This is to enable the participants to assess the validity of their beliefs and provide their own rationale for why the belief may not be true. This was done using both deductive and inductive reasoning. By applying deductive reasoning, the therapists asked the participants to discuss their several experiences regarding their indebtedness (e.g., having muscle tension, fear, anger) whenever the topic of loan indebtedness is brought up among colleagues. Using inductive reasoning, the therapists sought probable evidence and concluded that the participants who exhibit either or a combination of the experiences mentioned are suffering from an anxiety disorder. Representations of inductive reasoning include generalizations, extrapolations, and inferences.

#### 2.5.4. Section 4: Creating replacement beliefs

The theorist created an alternative replacement for beliefs participants hold about being indebted and exposed them to the knowledge of such replacements. Among alternatives created are alternative interpretations of events participants experienced (e.g., there is a need to be contemptuous with the much I have; I do not have to lead my life to please friends; I will let my spouse and children know my financial status; I should learn to face reality than living in pretense, etc). On the alternative perspectives for their own qualities and capabilities, the therapists encouraged the participants to decrease negative self-talk and redefine themselves in a more positive way that promotes more supportive behavior for success (e.g., the participants were allowed to think about their indebtedness, try to deescalate the thought and mentally create calm imagery which they can look up to, e.g., statuses of Jesus, Virgin Mary, Crucifixes and other images with inscriptions such as “it is well with me,” “relax for God is in control,” “tomorrow takes care of itself,” etc).

The therapists introduce relaxation techniques and mindfulness strategies to the participants as coping responses while trying to replace irrational beliefs. In relaxation techniques, participants were encouraged to incorporate strategies that change their mental anxiousness and anxiety each time they remember that they were indebted by changing their physical state, consciously relaxing tensed muscles, imagery, and using breathing exercises. In the mindfulness strategy, effort is concentrated on decreasing rumination and focusing on solutions. Using the diversion strategy, the therapists encouraged the participants to be mentally preoccupied and redirect their attention away from uncomfortable conditions that cannot be changed such as their loan indebtedness.

#### 2.5.5. Section 5: Modifying behavior

It is desirable for positive behavior to take the place of self-defeating behavior after irrational thoughts are replaced by more supportive alternative beliefs. The therapists focused on cognitive restructuring and helped participants explore the evidence confirming or refuting the identified irrational cognitions and their replacements. They adopted the use of exposure strategies as gradual steps of intense emersion of the participants. They used real and imagined stimuli techniques which involved role-playing, mental imagination, and acting “as if” to expose participants to more rational beliefs that helped them to modify their behavioral responses to the situation at hand (e.g., leading one’s life as though he is not the only person indebted with loan; assumption that loans helped others in the past execute big projects so mine will not be different; it is important for one to take care of himself and family than allowing other confuse him, etc).

The participants were encouraged to attend to previously avoided circumstances (e.g., meeting micro-finance loan administrators, discussion about being indebted), and were encouraged to observe and collect evidence either confirming or refuting their negative beliefs about indebted.

The use of the CBFT program helped to desensitize anxiety and counter irrational beliefs to developing behaviors that are more supportive for improving the participants’ overall quality of life, particularly mental health. After 10 weeks of treatment, participants in both groups were assessed at the second point to measure progress with improvement in the decrease of anxiety disorder using the measures.

A follow-up meeting was conducted after 2 months the expiration of the active treatment which lasted for 2 weeks owing to the participants’ tight schedules. During the follow-up stage, the researchers observed that the participants were able to notice their irrational beliefs about being indebted which led them to anxiety and replace them with more accurate and helpful beliefs. They were reportedly convinced that if they had not participated in the CBFT program, they would have had more serious mental problems order than anxiety. At the end of this meeting, the participants in treatment and waitlisted control groups completed the measurement tools.

Later, the participants in the waitlisted control group were invited to resume their own treatment sessions. They received the same treatment those in the active group received using the same time, manual, therapists, and venue. This was done since the participants in the waitlisted control group had the same problem, therefore, they had access to the treatment.

### 2.6. Intervention

CBFT is a 5-session treatment program designed to identify irrational beliefs, challenge irrational beliefs, test the validity of irrational beliefs, create replacement beliefs, and modify behavior among 74 employed workers with dysfunctional cognitive distortions and self-defeating beliefs that hinder or impede positive financial behavior. The duration for the CBFT program was 10 weeks, 1 session per 2 weeks for 90 minutes. CBFT according to Nabeshima and Klontz,^[55]^ was designed following the adoption of CBT for use in identifying, challenging, and changing problematic money scripts, which has been called a money script log. In essence, the money script log is a tool to help participants examine their thoughts, feelings, and unconscious thinking patterns around money. Participants were asked to identify specific financial situations, in which they feel some distress, identify the emotion, and then ask themselves. During the first session, introduction, therapeutic alliance with the participants, and identification of irrational beliefs about being indebted were established. Sessions 2 to 4 concentrated on challenging irrational beliefs, testing the validity of irrational beliefs, and creating replacement beliefs. In the final session, participants were taught how to modify irrational behavior. Several techniques were applied during sessions. Among those are relapse prevention techniques, cognitive restructuring, mood monitoring, problem-solving, self-management, and cognitive disputation.

### 2.7. Data analysis

The data from the pretest (before treatment), posttest (after treatment), and follow-up were subjected to statistical analysis using SPSS version 18. Specifically, 2-way analysis of covariance (ANCOVA) was used as a method of data analysis. ANCOVA was used to ascertain the effectiveness of CBFT in reducing anxiety among adult employees on loans determined using ANCOVA. The effect size of the intervention was reported using partial eta squared. The assumption of the homogeneity of variance was determined using Leven test of equality of variance (*F* = 0.649, *P* = .663 before treatment and *F* = 0.578, *P* = .717 after treatment for FAS-A; *F* = 0.470, *P* = .798 before treatment and *F* = 0.599, *P* = .700 after treatment for FAS-P).

## 3. Results

Table 2 reveals the mean and standard deviation outcomes for the participants in the treatment and control groups with respect to the length of loan as measured by FAS-A and FAS-P. At the pretest (before treatment) stage for CBFT, participants in 1 to 2 years, 3 to 4 years, and 5 years and above the length of the loan had mean financial anxiety scores of (X ® = 34.94, SD = 3.92), (X ® = 34.89, SD = 3.78), and (X ® = 36.77, SD = 3.17) as measured by FAS-A respectively. At the posttest (after treatment) stage, participants in 1 to 2 years, 3 to 4 years, and 5 years and above the length of the loan had mean financial anxiety scores of (X ® = 23.72, SD = 2.32), (X ® = 23.77, SD = 2.76), and (X ® = 22.23, SD = 2.62), respectively. At the follow-up stage, participants in 1 to 2 years, 3 to 4 years, and 5 years and above length of loan had mean financial anxiety scores of (X ® = 16.44, SD = 2.17), (X ® = 16.27, SD = 2.31), and (X ® = 16.40, SD = 2.41), respectively. The overall mean financial anxiety scores at the pretest, posttest, and follow-up stages for the CBFT group were 35.36, 23.37, and 16.37, respectively. The mean result presented in Table 2 indicates that there was a continuous decline in the mean financial anxiety scores of employees on loan in the treatment group over time. For the waitlisted control group (WCG) at the pretest stage, participants in 1 to 2 years, 3 to 4 years, and 5 years and above the length of the loan had mean financial anxiety scores of (X ® = 35.15, SD = 3.50), (X ® = 35.50, SD = 2.08), and (X ® = 32.91, SD = 3.81), respectively. At the posttest stage, participants in 1 to 2 years, 3 to 4 years, and 5 years and above length of the loan had mean financial anxiety scores of (X ® = 27.81, SD = 4.12), (X ® = 27.00, SD = 4.32), and (X ® = 28.18, SD = 2.44), respectively. At the follow-up stage, participants in 1 to 2 years, 3 to 4 years, and 5 years and above the length of loan had mean financial anxiety scores of (X ® = 25.11, SD = 3.33), (X ® = 24.62, SD = 5.23), and (X ® = 25.45, SD = 3.01), respectively. The overall mean financial anxiety scores at the pretest, posttest, and follow-up stages for the WCG were 35.68, 27.64, and 25.04, respectively. Although there was a continuous decline in the mean financial anxiety scores of employees on loan in the waitlisted group over time, it was not as sharp as that of the treatment group. The resulting base on the FAS by Prawitz et al was similar to that of the FAS by Archuleta et al, as shown in Table 2.

**Table 2:** Mean and standard deviation of participants in CBFT and WC groups with regards to length of loan on financial anxiety.

Length of loan	Number		Pretest		Posttest		Follow-up	
	CBFT	WCG	CBFT	WCG	CBFT	WCG	CBFT	WCG
	N_1_	N_2_	Mean (SD)	Mean (SD)	Mean (SD)	Mean (SD)	Mean (SD)	Mean (SD)
Financial Anxiety Scale, FAS-A								
1–2 yr	18	26	34.94 (3.92)	35.15 (3.50)	23.72 (2.32)	27.81 (4.12)	16.44 (2.17)	25.11 (3.33)
3–4 yr	22	16	34.89 (3.78)	35.50 (2.80)	23.77 (2.76)	27.00 (4.32)	16.27 (2.31)	24.62 (5.23)
5 yr and above	13	11	36.77 (3.17)	32.91 (3.81)	22.23 (2.62)	28.18 (2.44)	16.40 (2.41)	25.45 (3.01)
Total	53	53	35.36 (3.71)	34.79 (3.45)	23.37 (2.67)	27.64 (3.86)	16.37 (2.25)	25.04 (3.88)
Financial Anxiety Scale, FAS-P								
1–2 yr	18	26	33.50 (4.36)	33.73 (3.54)	21.91 (2.04)	27.80 (2.40)	19.39 (3.09)	21.64 (1.44)
3–4 yr	22	16	32.73 (3.71)	34.50 (4.65)	22.55 (2.59)	27.47 (2.17)	19.23 (3.65)	22.53 (2.80)
5 yr and above	13	11	33.38 (3.18)	32.45 (3.96)	23.23 (2.80	27.45 (2.62)	21.54 (4.29)	21.55 (1.86)
Total	53	53	33.15 (3.77)	33.70 (3.97)	22.51 (2.48)	27.63 (2.34)	20.06 (3.68)	21.88 (2.02

CBFT = cognitive behavioral financial therapy, FAS-A = Financial Anxiety Scale by Archuleta et al, FAS-P = Financial Anxiety Scale by Prawitz et al, N_1_ = number of participants in CBFT group, N_2_ = number of participants in WCG group, WCG = waitlist control group.

Table 3 reveals the main effect due to CBFT, main effect due to the length of the loan, and interaction effect of groups and length of loan with respect to financial anxiety. For main effect due to CBFT outcomes for the participants in the treatment group (CBFT) compared to the WCG over the 3 periods indicates that before the treatment, there was no significant difference between the treatment and control groups in initial mean financial anxiety score among employees on loan in Nigeria as measured by FAS-A, *F*(1,105) = 1.962, *P* = .164, $\eta_{p}^{2}=0.019$, *∆R*^2^ = 0.023. At the posttreatment, the effect of CBFT was significant in reducing the mean financial anxiety score among employees on loan in Nigeria as measured by FAS-A, *F*(1,105) = 43.349, *P* = .001, $\eta_{p}^{2}=0.302$, *∆R*^2^ = 0.285. After the posttreatment, a follow-up result shows that *F*(1,105) = 176.129, *P* = .001, $\eta_{p}^{2}=0.638$, *∆R*^2^ = 0.640. The $\eta_{p}^{2}$ (partial eta squared) values or effect size of 0.302 and 0.638 at posttreatment and follow-up levels indicate that CBFT accounted for a 30.2% and 63.8% reduction in financial anxiety value in Nigeria, respectively. This result is with regard to the main effect due to CBFT for FAS-A. In terms of length of loan, before the treatment, after the posttreatment, and follow-up levels, Table 3 reveals that there was no significant difference among employees on loans from various length of loan in the mean financial anxiety score in Nigeria as measured by FAS-A, *F*(1,105) = 0.068, *P* = .934, $\eta_{p}^{2}=0.001$; *F*(1,105) = 0.252, *P* = .778, $\eta_{p}^{2}=0.005$; and *F*(1,105) = 0.202, *P* = .818, $\eta_{p}^{2}=0.004$, respectively. The $\eta_{p}^{2}$ (partial eta squared) values or effect size of 0.005 and 0.004 at posttreatment and follow-up levels indicate that CBFT accounted for 0.5% and 0.4% reduction in financial anxiety among employees on loan in Nigeria, respectively. Concerning interaction effect of groups and length of loan after the posttreatment and follow-up levels, Table 3 reveals that there was no significant interaction effect in the mean financial anxiety score among employees on loan in Nigeria as measured by FAS-A, *F*(1,105) = 1.238, *P* = .288, $\eta_{p}^{2}=0.024$; and *F*(1,105) = 0.073, *P* = .930, $\eta_{p}^{2}=0.001$, respectively. The $\eta_{p}^{2}$ (partial eta squared) values or effect size of 0.024 and 0.001 at posttreatment and follow-up levels indicate that CBFT accounted for 2.4% and 0.1% reduction in financial anxiety among employees on loan in Nigeria, respectively.

**Table 3:** Effect of CBFT on financial anxiety as measured by FAS-A.

Source	Dependent variable	Type III sum of squares	df	Mean square	*F*	Sig.	Partial eta squared
Corrected model	Pretest	93.259*	5	18.652	1.490	0.200	0.069
	Posttest	515.033†	5	103.007	9.386	0.000	0.319
	Follow-up	1992.764‡	5	398.553	38.353	0.000	0.657
Intercept	Pretest	1.194E5	1	1.194E5	9536.639	0.000	0.990
	Posttest	63,065.714	1	63,065.714	5746.530	0.000	0.983
	Follow-up	41,829.754	1	41,829.754	4025.304	0.000	0.976
Groups	Pretest	24.571	1	24.571	1.962	0.164	0.019
	Posttest	475.734	1	475.734	43.349	0.000	0.302
	Follow-up	1830.278	1	1830.278	176.129	0.000	0.638
LOL	Pretest	1.703	2	0.852	0.068	0.934	0.001
	Posttest	5.521	2	2.761	0.252	0.778	0.005
	Follow-up	4.195	2	2.097	0.202	0.818	0.004
Groups × LOL	Pretest	84.440	2	42.220	3.372	0.038	0.063
	Posttest	27.172	2	13.586	1.238	0.294	0.024
	Follow-up	1.517	2	0.759	0.073	0.930	0.001
Error	Pretest	1252.137	100	12.521			
	Posttest	1097.457	100	10.975			
	Follow-up	1039.170	100	10.392			
Total	Pretest	1.318E5	106				
	Posttest	70,590.000	106				
	Follow-up	48,485.000	106				
Corrected total	Pretest	1345.396	105				
	Posttest	1612.491	105				
	Follow-up	3031.934	105				

α = 0.05.

CBFT = cognitive behavioral financial therapy, FAS-A = Financial Anxiety Scale by Archuleta et al, LOL = length of loan.

* *R*^2^ = 0.069 (adjusted *R*^2^ = 0.023).

† *R*^2^ = 0.319 (adjusted *R*^2^ = 0.285).

‡ *R*^2^ = 0.657 (adjusted *R*^2^ = 0.640).

Table 4 reveals the main effect due to CBFT, main effect due to length of loan, and interaction effect groups and length of loan with respect to financial anxiety. For main effect due to CBFT outcomes for the participants in the treatment group (CBFT) compared to the WCG over the 3 periods indicates that before the treatment, there was no significant difference between the treatment and control groups in the initial mean financial anxiety score among employees on loan in Nigeria as measured by FAS-P, *F*(1,105) = 0.204, *P* = .652, $\eta_{p}^{2}=0.002$, *∆R*^2^ = 0.022. At the posttreatment, the effect of CBFT was significant in reducing the mean financial anxiety score among employees on loan in Nigeria as measured by FAS-P, *F*(1,105) = 99.448, *P* = .001, $\eta_{p}^{2}=0.499$, *∆R*^2^ = 0.516. After the posttreatment, a follow-up result show that *F*(1,105) = 9.415, *P* = .003, $\eta_{p}^{2}=0.086$, *∆R*^2^ = 0.108. The $\eta_{p}^{2}$ (partial eta squared) values or effect size of 0.499 and 0.108 at posttreatment and follow-up levels indicate that CBFT accounted for 49.9% and 8.6% reduction in financial anxiety value in Nigeria, respectively. This result is with respect to main effect due to CBFT for FAS-P. In terms of length of loan, before the treatment, after the posttreatment, and follow-up levels, Table 3 reveals that there was no significant difference among employees on loan with various length of loan in the mean financial anxiety score in Nigeria as measured by FAS-P, *F*(1,105) = 0.290, *P* = .749, $\eta_{p}^{2}=0.006$; *F*(1,105) = 0.137, *P* = .872, $\eta_{p}^{2}=0.003$; and *F*(1,105) = 0.650, *P* = .524, $\eta_{p}^{2}=0.013$, respectively. The $\eta_{p}^{2}$ (partial eta squared) values or effect size of 0.003 and 0.013 at posttreatment and follow-up levels indicate that CBFT accounted for 0.3% and 1.3% reduction in financial anxiety among employees on loan in Nigeria, respectively. Concerning interaction effect of groups and length of loan after the posttreatment and follow-up levels, Table 3 reveals that there was no significant interaction effect in the mean financial anxiety score among participants in Nigeria as measured by FAS-P, *F*(1,105) = 1.046, *P* = .355, $\eta_{p}^{2}=0.020$ and *F*(1,105) = 2.067, *P* = .132, $\eta_{p}^{2}=0.040$, respectively. The $\eta_{p}^{2}$ (partial eta squared) values or effect size of 0.020 and 0.040 at posttreatment and follow-up levels indicate that CBFT accounted for 2.0% and 4.0% reduction in financial anxiety among employees on loan in Nigeria, respectively.

**Table 4:** Effect of CBFT on financial anxiety as measured by FAS-P.

Source	Dependent variable	Type III sum of squares	df	Mean square	*F*	Sig.	Partial eta squared
Corrected model	Pretest	42.113*	5	8.423	0.552	0.736	0.027
	Posttest	781.810†	5	156.362	23.371	0.000	0.539
	Follow-up	188.748‡	5	37.750	3.541	0.005	0.150
Intercept	Pretest	108,487.173	1	108,487.173	7110.261	0.000	0.986
	Posttest	61,760.888	1	61,760.888	9231.406	0.000	0.989
	Follow-up	43,891.570	1	43,891.570	4116.653	0.000	0.976
Groups	Pretest	3.116	1	3.116	0.204	0.652	0.002
	Posttest	665.337	1	665.337	99.448	0.000	0.499
	Follow-up	100.382	1	100.382	9.415	0.003	0.086
LOL	Pretest	8.859	2	4.430	0.290	0.749	0.006
	Posttest	1.837	2	0.919	0.137	0.872	0.003
	Follow-up	13.856	2	6.928	0.650	0.524	0.013
Groups × LOL	Pretest	27.955	2	13.977	0.916	0.403	0.018
	Posttest	13.995	2	6.998	1.046	0.355	0.020
	Follow-up	44.076	2	22.038	2.067	0.132	0.040
Error	Pretest	1525.783	100	15.258			
	Posttest	669.030	100	6.690			
	Follow-up	1066.196	100	10.662			
Total	Pretest	119,991.000	106				
	Posttest	68,755.000	106				
	Follow-up	48,760.000	106				
Corrected total	Pretest	1567.896	105				
	Posttest	1450.840	105				
	Follow-up	1254.943	105				

α = 0.05.

CBFT = cognitive behavioral financial therapy, FAS-P = Financial Anxiety Scale by Prawitz et al, LOL = length of loan.

* *R*^2^ = 0.027 (adjusted *R*^2^ = −0.022).

† *R*^2^ = 0.539 (adjusted *R*^2^ = 0.516).

‡ *R*^2^ = 0.150 (adjusted *R*^2^ = 0.108).

## 4. Discussion

The results showed that the effect of CBFT was significant in reducing the mean financial anxiety score among employees on loans. Also, the findings revealed a sustained significant impact of CBFT on anxiety among employees on loan who were exposed to treatment compared to those in the waitlisted control group at end of the intervention. The result is consistent with the findings of a number of studies that incorporated mental health and financial interventions. Firstly, the study conducted by previous studies^[67–69]^ on which participants reported reductions in psychological distress, anxiety, and worry about money and finance-related situations after a treatment approach that integrated psychological and financial concepts to treat unhealthy financial behaviors and related psychological symptomology in a 6-day experiential therapy program. Second is a pilot study by Gale et al^[70]^ which looked at the effectiveness of a financial planner and a family therapist working together to provide therapy for 12 couples experiencing concomitant financial and relational distress. In the utilization of the sessions treatment program in the current study, the researchers found overwhelmingly that the couples reported fewer relationship problems, less financial strain, and improved financial well-being.

In the reviewed studies, therapists from both the mental health and financial fields need to be present in the sessions for effective results. This is not always a viable option in therapists’ work due to scheduling or monetary constraints. Many times, mental health and financial therapists collaborate or consult each other in working with people who are experiencing both financial and other mental health disorders such as marital stress, anxiety, depression, and other psychological issues. Thus, there is a need for an approach that will integrate both mental health and financial fields’ experiences or knowledge so that, individually, therapists can utilize them to resolve interrelated topics in session. The researchers found that CBFT, being an aspect of CBT can bridge this gap.

CBT is a problem-focused and action-oriented form of therapy, meaning it is used to treat specific problems related to a diagnosed mental disorder.^[52]^ The overall goal of treatment with CBT is symptom reduction, improvement in functioning, and remission of mental disorder by teaching new information-processing skills and coping mechanisms to individuals with mental health problems occasioned by irrational or dysfunctional beliefs.^[50,53]^ CBT has an impact on a good number of mental health conditions associated with anxiety.^[71,72]^ This can be observed in the concordance of the current findings with the outcomes from other studies using CBT on samples of adults with anxiety and other mental disorders. For instance, studies revealed the effectiveness of CBT in the treatment of adults with anxiety disorders^[54,71]^ and mood disorders.^[73]^

The findings revealed a significant reduction in anxiety disorder among employees on loan who were exposed to the CBFT program group compared with their counterparts in the waitlisted control group at the end of the intervention. The significant reduction in anxiety disorder observed among participants in the treatment group could be attributed to the roles the therapists played in assisting the clients in finding and practicing effective strategies that addressed the identified irrational beliefs and behaviors, which was not so with participants in the waitlisted control group. This has helped to decrease symptoms of anxiety disorder as observed by the researchers toward the end of the intervention with CBFT. In particular, if people often experience significant financial anxiety or similar affective states, without interventions such as the use of CBFT, they may tend to bias their attention, belief, or appraisals toward adverse information, amplifying concerns and anxieties about their finances.^[74]^ Grable et al^[75]^ are of the view that registered anxiety could make individuals feel reluctant to contemplate and address their financial challenges, thus, compounding their anxiety levels.

The finding supports other studies on cognitive and behavioral interventions, which have been shown to be effective in reducing financially-related stressors and behaviors,^[76–78]^ and maladaptive financial beliefs and behaviors.^[68]^ CBT was also found as an effective method of preventing or delaying relapses.^[79,80]^

Similarly, other considerable numbers of studies have also reported the effectiveness of CBT in the reduction of psychological problems associated with core thoughts. This is as Field et al^[52]^ maintained that CBT is a therapeutic intervention that plays a useful role in cushioning the effects of psychological disorders. An interoceptive exposure for the treatment of panic disorder using CBT was moderately effective and superior to control/pill placebo treatments and applied relaxation.^[81,82]^

In a follow-up study, the analysis of variance test as shown in Table 4 revealed a sustained significant impact of CBFT on the employees in the CBFT program group after 2 months compared to employees in the waitlisted control group. The finding is in tune with the findings of a study on the treatment of social anxiety disorder, in which CBT showed a moderate to large effect size at immediate posttreatment and follow-up.^[83]^ The finding is also consistent with the findings of Ede et al^[84]^ which the posttreatment and follow-up measures, found the efficacy of rational-emotive health therapy on the reduction of parenting stress among parents of children with autism spectrum disorders was significant. Egenti et al^[85]^ in a study on randomized controlled evaluation of the effect of music therapy with CBT on social anxiety symptoms at follow-up assessment after 3 months, found a significant reduction in social anxiety disorder for the participants in the treatment group.

### 4.1. Practice implications

The results of this study suggest implications for further research and practice. For instance, CBFT has implications for practitioners working with financial and family issues. Cognitive financial therapists, family and marriage counselors, psychologists, mental health professionals, and financial educators can use CBFT to identify and help individuals and couples who present financial issues as a source of their worries and maladjusted beliefs and behaviors. There could be collaboration among the above-mentioned experts in terms of referrals and consultation in addressing underlying issues associated with beliefs and behavior, among which are family dynamics, anxiety, depression, ideation, and other psychological issues. Looking beyond a collaboration is the development of an intervention program to identify and diagnose distorted or maladaptive financial beliefs and behavior.

Other researchers and clinicians can collaborate to identify strategies and evidence-based practices to allow cognitive financial therapists and other financial and mental health practitioners a way to work with their clients effectively. Also, there is a need for future studies to both replicate and extend the findings from this study. Cognitive behavioral therapists should recognize the emotional behaviors that people who are indebted with loans might display. Letting employees on loans and those intending to obtain loans about the availability of cognitive behavioral therapists and mental health services, should they begin feeling anxious about their debts before they impact their work life, could help keep them safe. By recognizing that incurred loan debt can cause anxiety disorder among individuals, cognitive behavioral financial therapists can play a role in helping those who have experienced financial anxiety cope with the experience and make sure that lingering cognitive behavioral changes do not have a harmful effect on one’s financial health.

### 4.2. Implications for anthropological philosophers

Philosophical anthropologists should examine how workers’ experiences of anxiety are influenced by the human condition, especially our jobs. According to this viewpoint, employees’ anxiety is a serious crisis of meaning connected to certain aspects of the contemporary workplace rather than just a psychiatric condition. In older societies, work was often closely related to a sense of community or survival. Profit and performance indicators in the modern workplace can mask a deeper sense of purpose. Employee discomfort and burnout may result from this “existential vacuum,” where their employment has no meaningful relationship to their personal values and aspirations.

Existentialists like Søren Kierkegaard believe that anxiety is the weight of being accountable for one’s decisions and the dizziness of freedom. This freedom and the institutional restraints of the job are constantly at odds in the workplace. Living a genuine existence in line with one’s true self is emphasized by existentialism. However, the workplace may force people to put on a “working façade” or take on social roles that don’t feel authentic. This dissonance, known as “working in dissonance,” causes anxiety as the worker feels like they are losing their true selves at work.

According to philosophical anthropology, workers’ anxiety disorders in the workplace reflect a profound human crisis regarding contemporary work. It shows how a purely instrumental, results-driven workplace culture stifles people’s search for purpose. The innate human potential for autonomy is being suppressed by institutional limitations and impractical demands. Pressures to conform to toxic standards and inauthentic roles undermine the human spirit’s need for authenticity and growth.

Spanish philosopher Leonardo Polo believes that the goal of labor is the development of each individual involved in productive and organized activity.^[86]^ Therefore, it is essential that organizational charts and procedures are designed with this goal in mind, and those responsible for creating and altering them should consider this.

### 4.3. Strengths of the study

The strengths of this study include the sampling procedures that provided a large representation of employees across major Nigerian workforce, the ability to control for important confounding factors, the use of 3 different well-validated questionnaires to measure anxiety disorder as against the 1-item measurement of anxiety used repeatedly in studies; and the employment of follow-up exercise after 2 months of treatment and posttest assignment. In a final point, the researchers discovered the dearth of studies on the impact of CBFT on maladaptive financial beliefs, particularly as it relates to being indebted.

### 4.4. Limitations of the study

The main drawback that could have influenced the outcomes of this study is the generalizability of the sample. Being that the study concentrated on employees on loan within a specified period, the relatively small sample size limited the generalization of the findings to other populations in different sectors that might have similar anxiety disorders. Second, the sample consisted of employees who indicated interest in participating in the intervention exercise and who may have been experiencing above-normal levels of anxiety in regard to their loan indebtedness compared with the average employee on a loan. It would be worthwhile to carry out further longitudinal research with well-validated instruments of mental disorders such as depression, stress, worries, irritability, etc, as well as detailed assessments of debt, looking at onset, amount, and source order from microfinance banks.

## 5. Conclusion

Generally, financial anxiety is a nonclinical and public health issue in Nigeria and among employees in particular. Based on this, we tested the efficacy of CBFT in reducing anxiety related to indebtedness. Also, this study is the first to report the impacts of CBFT on anxiety among employees on loan, especially in low-income countries like Nigeria. Conclusively, CBFT is a useful psychotherapeutic intervention for the reduction of anxiety among employees on loan. Given the usefulness of this intervention, we recommend that future researchers in the field of cognitive-behavioral approaches utilize and apply it in their professional practices.

## Acknowledgments

We sincerely appreciate all our participants who showed a high sense of commitment from the beginning of the program till the end.

## Author contributions

**Conceptualization:** Sebastian Okechukwu Onah, Queen E. Igabari, Uchechukwu Hope Ekwueme, Fedrick C. Onah, Moses Onyemaechi Ede, Ogochukwu Vivian Nwabuani, Michael Chugozie Anyaeihe.

**Data curation:** Sebastian Okechukwu Onah, Queen E. Igabari, Uchechukwu Hope Ekwueme, Moses Onyemaechi Ede, Ogochukwu Vivian Nwabuani, Obiageli Loretta Aniaku.

**Formal analysis:** Queen E. Igabari, Uchechukwu Hope Ekwueme, Kelechi Ruth Ede, Moses Onyemaechi Ede, Ogochukwu Vivian Nwabuani.

**Funding acquisition:** Sebastian Okechukwu Onah, Queen E. Igabari, Uchechukwu Hope Ekwueme, Kelechi Ruth Ede, Fedrick C. Onah, Moses Onyemaechi Ede, Ifeyinwa Manafa, Ogochukwu Vivian Nwabuani, Obiageli Loretta Aniaku, Michael Chugozie Anyaeihe, George Ohabuenyi Abah, Innocent I. Enweh, Monday Ume Nwodo, Ndubuisi Collins Enyi.

**Investigation:** Sebastian Okechukwu Onah, Uchechukwu Hope Ekwueme, Kelechi Ruth Ede, Fedrick C. Onah, Moses Onyemaechi Ede, Ifeyinwa Manafa, Ogochukwu Vivian Nwabuani, Michael Chugozie Anyaeihe, George Ohabuenyi Abah, Innocent I. Enweh, Ndubuisi Collins Enyi.

**Methodology:** Sebastian Okechukwu Onah, Queen E. Igabari, Uchechukwu Hope Ekwueme, Kelechi Ruth Ede, Fedrick C. Onah, Moses Onyemaechi Ede, Ifeyinwa Manafa, Ogochukwu Vivian Nwabuani, Obiageli Loretta Aniaku, Michael Chugozie Anyaeihe, George Ohabuenyi Abah.

**Project administration:** Sebastian Okechukwu Onah, Uchechukwu Hope Ekwueme, Kelechi Ruth Ede, Fedrick C. Onah, Moses Onyemaechi Ede, Ifeyinwa Manafa, Ogochukwu Vivian Nwabuani, Michael Chugozie Anyaeihe, George Ohabuenyi Abah, Innocent I. Enweh, Ndubuisi Collins Enyi.

**Resources:** Sebastian Okechukwu Onah, Queen E. Igabari, Uchechukwu Hope Ekwueme, Kelechi Ruth Ede, Fedrick C. Onah, Moses Onyemaechi Ede, Ifeyinwa Manafa, Ogochukwu Vivian Nwabuani, Obiageli Loretta Aniaku, Michael Chugozie Anyaeihe, Monday Ume Nwodo, Ndubuisi Collins Enyi.

**Software:** Sebastian Okechukwu Onah, Queen E. Igabari, Uchechukwu Hope Ekwueme, Kelechi Ruth Ede, Fedrick C. Onah, Moses Onyemaechi Ede, Ifeyinwa Manafa, Ogochukwu Vivian Nwabuani, Obiageli Loretta Aniaku, Michael Chugozie Anyaeihe, Monday Ume Nwodo, Ndubuisi Collins Enyi.

**Supervision:** Sebastian Okechukwu Onah, Queen E. Igabari, Uchechukwu Hope Ekwueme, Kelechi Ruth Ede, Fedrick C. Onah, Moses Onyemaechi Ede, Ifeyinwa Manafa, Ogochukwu Vivian Nwabuani, Obiageli Loretta Aniaku, Michael Chugozie Anyaeihe, George Ohabuenyi Abah, Innocent I. Enweh, Monday Ume Nwodo, Ndubuisi Collins Enyi.

**Validation:** Sebastian Okechukwu Onah, Queen E. Igabari, Uchechukwu Hope Ekwueme, Kelechi Ruth Ede, Fedrick C. Onah, Moses Onyemaechi Ede, Ifeyinwa Manafa, Ogochukwu Vivian Nwabuani, Obiageli Loretta Aniaku, Michael Chugozie Anyaeihe, George Ohabuenyi Abah, Innocent I. Enweh, Monday Ume Nwodo, Ndubuisi Collins Enyi.

**Visualization:** Sebastian Okechukwu Onah, Queen E. Igabari, Uchechukwu Hope Ekwueme, Fedrick C. Onah, Moses Onyemaechi Ede, Ifeyinwa Manafa, Ogochukwu Vivian Nwabuani, Obiageli Loretta Aniaku, Michael Chugozie Anyaeihe, George Ohabuenyi Abah, Innocent I. Enweh, Monday Ume Nwodo, Ndubuisi Collins Enyi.

**Writing – original draft:** Sebastian Okechukwu Onah, Uchechukwu Hope Ekwueme, Kelechi Ruth Ede, Fedrick C. Onah, Moses Onyemaechi Ede, Ogochukwu Vivian Nwabuani, Michael Chugozie Anyaeihe.

**Writing – review & editing:** Sebastian Okechukwu Onah, Queen E. Igabari, Uchechukwu Hope Ekwueme, Fedrick C. Onah, Moses Onyemaechi Ede, Ifeyinwa Manafa, Ogochukwu Vivian Nwabuani, Obiageli Loretta Aniaku, George Ohabuenyi Abah, Innocent I. Enweh, Ndubuisi Collins Enyi.
